# Comparison of upper sublethal and lethal temperatures in three species of rice planthoppers

**DOI:** 10.1038/s41598-019-52034-7

**Published:** 2019-11-07

**Authors:** Shahbaz Ali, Pei Li, Asad Ali, Maolin Hou

**Affiliations:** 1 State Key Laboratory for Biology of Plant Diseases and Insect Pests, Institute of Plant Protection, Chinese Academy of Agricultural Sciences, Beijing, 100193 China; 2 Scientific Observing and Experimental Station of Crop Pests in Guilin, Ministry of Agriculture, Guilin, 541399 China; 3 Southern Regional Collaborative Innovation Center for Grain and Oil Crops in China, Changsha, 410128 China

**Keywords:** Animal behaviour, Animal behaviour, Animal behaviour, Entomology, Entomology

## Abstract

Temperature is an important environmental factor for ectotherms’ fitness and survival. The upper sublethal and lethal temperatures were compared between adults of three closely related destructive planthopper species, the small brown planthopper (*Laodelphax striatellus*, SBPH), the brown planthopper (*Nilaparvata lugens*, BPH), and the white-backed planthopper (*Sogatella furcifera*, WBPH) in the absence and presence of the host plant (*Oryza sativa*, var. Taichong1). Values of the critical thermal maxima (CT_max_) were higher in SBPH than in both BPH and WBPH and higher in BPH than in WBPH, and values of the heat coma temperatures (HCT) were higher in both BPH and SBPH than in WBPH. CT_max_ and HCT values were higher in the presence than in the absence of plant material. Between sexes, females generally showed higher CT_max_ and HCT than males. The upper lethal temperatures (ULT_50_) measured in the absence of plant material were not significantly different among the planthopper species. The planthoppers also exhibited different behaviors in an increasing temperature regime, with fewer insects dropping-off from the plant in SBPH than in BPH and WBPH. These results indicate that SBPH and BPH are more heat tolerant than WBPH. The findings highlight the biological divergence in closely related planthopper species and the importance of performing the heat tolerance measurement in an ecologically relevant setting, which serves to predict seasonal and spatial occurrence patterns of the destructive planthopper species.

## Introduction

Insects are ectotherms and are of limited ability to regulate body temperature above and below optimum. Consequently, temperature can play a central role in insect development^1^, behavior^2^, and seasonality and distribution^3–5^. Therefore, thermal tolerance traits are usually examined in the laboratory to forecast ectotherms’ field success and pest outbreaks.

In thermal tolerance studies of insects, sub-lethal and lethal traits are usually characterized. Critical thermal limit, coma temperature and coma recovery times are the sub-lethal traits that are measured before the death of the organism. In contrast, lethal temperature and lethal time are parameters used to indicate lethal traits, where mortality is induced in the exposed organism^6^. When an insect is exposed to increasing temperatures for a certain duration, different observable and measurable events occur^7^: when the exposed organism shows uncoordinated movement until motionless, it reaches the critical thermal maximum (CT_max_); when the appendages (legs and antennae) are incapable of movement and the organism enters a ‘heat coma’ state, it comes to the heat coma temperature (HCT)^8^. At even higher temperatures, the insect dies, and the temperature is the upper lethal temperature (ULT)^7^. ULT_50_ is the upper high temperature that results in 50% mortality in a sample population. Estimating these parameters offers a physiological insight into the events that are ecologically important^8^. For instance, insects at CT_max_ are incapable of moving move and are therefore unable to find food sources or escape from natural enemies^1^; and to a wider extent, this can affect insects’ distribution and potential range expansion^3,9^.

Rice is the staple food for one third of the world’s population and is attacked by three closely related destructive planthopper species: the small brown planthopper (SBPH) *Laodelphax striatellus* (Fallén), the brown planthopper (BPH) *Nilaparvata lugens* (Stål), and the white-backed planthopper (WBPH) *Sogatella furcifera* (Horvath) (Hemiptera: Delphacidae). The three species are similar in morphology and exhibit wing-dimorphism in the adult stage. They cause damage to rice plants directly by sucking phloem sap and indirectly by transmitting plant viruses^10^. However, the three planthopper species exhibit different tolerance to low temperatures; WBPH and BPH cannot survive 0 °C for 5 days^10^ while SBPH can overwinter locally at sub-zero temperatures^11^. WBPH and BPH can overwinter only in areas to the south of 21–25°N and occurrence in the northern areas depends on migration from the south, principally the Indo-China Peninsula. The planthopper species also differ in spatial distribution. WBPH and BPH can occur from the tropics to 42–44°N in Asia through migration, while SBPH occurs primarily in the temperate regions^10^. The temperatures of July and August in the subtropics during 1985–2010 average 27.3 °C and 27.0 °C, respectively, while in the temperate regions they are 28.8 °C and 28.0 °C, respectively, in China (data calculated from the Chinese Meteorological Data Sharing Service System, http://cdc.cma.gov.cn). Further, the three planthopper species also differ in host range. The three species all flourish on rice, but BPH is monophagous and WBPH and SBPH are polyphagous on rice, wheat, corn and other plants^10^. The planthopper species also show varying performance at high temperatures. An analysis of time-temperature-mortality models at constant temperatures between 35 and 40 °C revealed longer lethal time in SBPH than in WBPH and BPH^12^. Further tests employing varying high temperatures showed reduced nymphal duration, adult longevity and fecundity in WBPH and BPH in comparison with SBPH^13^. Piyaphongkul *et al*.^8^ measured CT_max_, HCT and ULT of BPH and found that thermal tolerance differed between developmental stages. Furthermore, they indicated that acclimation increases heat tolerance in BPH^14^. Using transcriptional analysis, Huang *et al*.^15^ found temperature modulation of genes related to thermal tolerance in the three planthopper species and suggested that the differentially expressed genes of heat shock proteins in the three planthopper species at high temperature might contribute to their different capacities for heat tolerance. However, the differences in upper sublethal and lethal temperatures between the three planthopper species remain unaddressed, especially considering that the mid-latitude species seem most prone to experience heat stress^4^.

Thermal tolerance has mostly been measured in the absence of the host plant, which may be problematic for phloem-feeding insects^16^. Host plant contact is reported to enhance aphids’ cold tolerance, i.e., lethal temperature^17^, lethal time^18^ and CT_min_^16^. These results question the validity of measuring heat tolerance in the absence of the host plant for predictions and decisions concerning pest species (e.g., timing and severity of pest outbreaks, efficiency and establishment potential of invasive species, implications of climate change)^16^.

In this study, we hypothesize that the three closely related planthopper species differ in their heat tolerance and that contact with host plant can increase their ability to withstand heat stress. Heat tolerance was measured in terms of CT_max_, HCT and ULT in both the absence and presence of their common host plant, which are expected to obtain results of ecological relevance to the field conditions. The current results will further our understanding of the spatio-temporal occurrence patterns of the three planthopper species in relation to their heat tolerance.

## Results

### CT_max_

**Figure 1:**
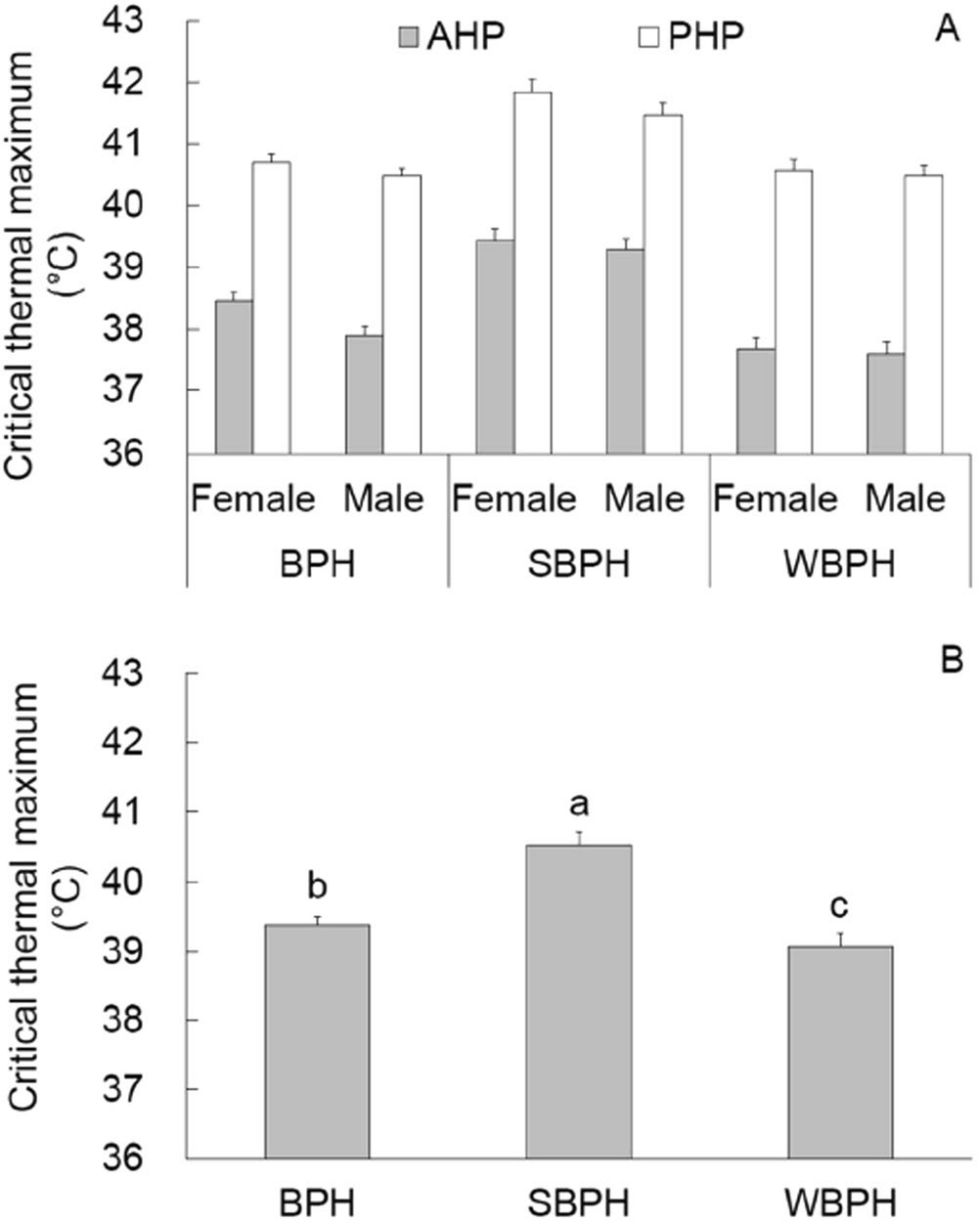
Critical thermal maximum (mean ± SE) of adults of the three species of planthopper. (**A**) All combinations of planthopper species, planthopper sexes and plant material (n = 40), (**B**) Main effect of planthopper species (n = 160).

In the absence of plant material, the CT_max_ ranged between 37.5–39.5 °C, while in the presence of plant material, between 40.5–42.0 °C (Fig. 1A). Significant influence on CT_max_ was observed for planthopper species (*F* = 182.3, df = 2,480, *P* < 0.001), planthopper sex (*F* = 7.3, df = 1,480,, *P* = 0.014), plant material (*F* = 773.1, df = 1,480, *P* < 0.001), and the interaction between planthopper species and plant material (*F* = 8.1, df = 2,480, *P* = 0.038). Between the planthopper species, mean CT_max_ values were higher in SBPH than in both BPH and WBPH (Tukey test, *P* < 0.001), and higher in BPH than in WBPH (Tukey test, *P* = 0.038; Fig. 1B). CT_max_ values were significantly higher in the presence than in the absence of plant material and higher in the females than in the males.

**Figure 2:**
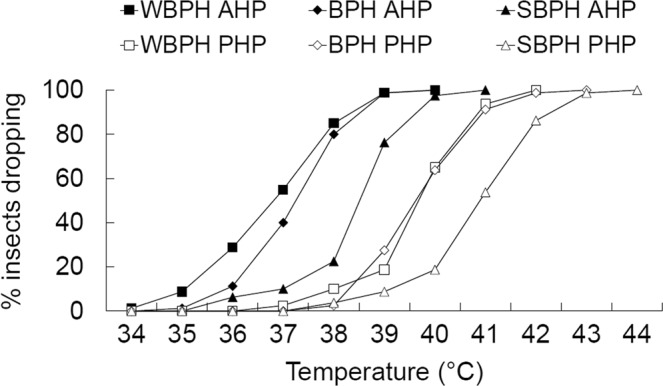
Cumulative percentage of planthopper adults of three species dropping off in the absence (AHP, in solid bullet) and in the presence (PHP, in hollow bullet) of host plant when subjected to an increasing temperature regime from 26 °C to 35 °C at 0.5 °C/min and then from 35 °C to 50 °C at 0.1 °C/min. n = 80.

When subjected to an increasing temperature regime, the insects exhibited different behaviors, i.e., dropping off or remaining on the tube wall or rice stem. Irrespective of species, proportions of insect dropping (Fig. 2) were found to be higher in the absence than in the presence of plant material at the 11 tested temperatures except 34 and 44 °C (*χ*^2^ ≥ 7.104, *P* ≤ 0.008). Between the species, proportions of insects dropping were different at seven out of the 11 tested temperatures (*χ*^2^ ≥ 7.120, *P* ≤ 0.028), with fewer insects dropping in SBPH. Regression of the cumulative proportions of insects dropping to temperatures (Table 1) showed that the temperatures with 50% insects dropping were 39.0, 40.4 and 38.9 °C for BPH, SBPH and WBPH, respectively, in the absence of plant material and 42.0, 43.0 and 41.3 °C for BPH, SBPH and WBPH, respectively, in the presence of plant material (Fig. 2).

**Table 1:** Results of logistic regression analysis of cumulative proportions of insects dropping-off in response to increasing temperatures.

Species	Plant	Coefficient of regression equation			*R* ^2^	ANOVA		
		Term	Value	*P*		*F*	df	*P*
BPH	Absence	Constant	1.284E12	0.901	0.811	17.176	1, 5	0.014
		Temperature	0.435	0.008				
	Presence	Constant	5.987E9	0.912	0.705	9.571	1, 5	0.036
		Temperature	0.524	0.009				
SBPH	Absence	Constant	3.317E9	0.859	0.817	22.262	1, 6	0.005
		Temperature	0.506	0.001				
	Presence	Constant	3.397E11	0.829	0.922	47.187	1, 5	0.002
		Temperature	0.47	0.001				
WBPH	Absence	Constant	9.252E8	0.789	0.922	47.612	1, 5	0.002
		Temperature	0.535	<0.001				
	Presence	Constant	6.67E8	0.782	0.906	48.039	1, 6	0.001
		Temperature	0.56	<0.001				

### HCT

**Figure 3:**
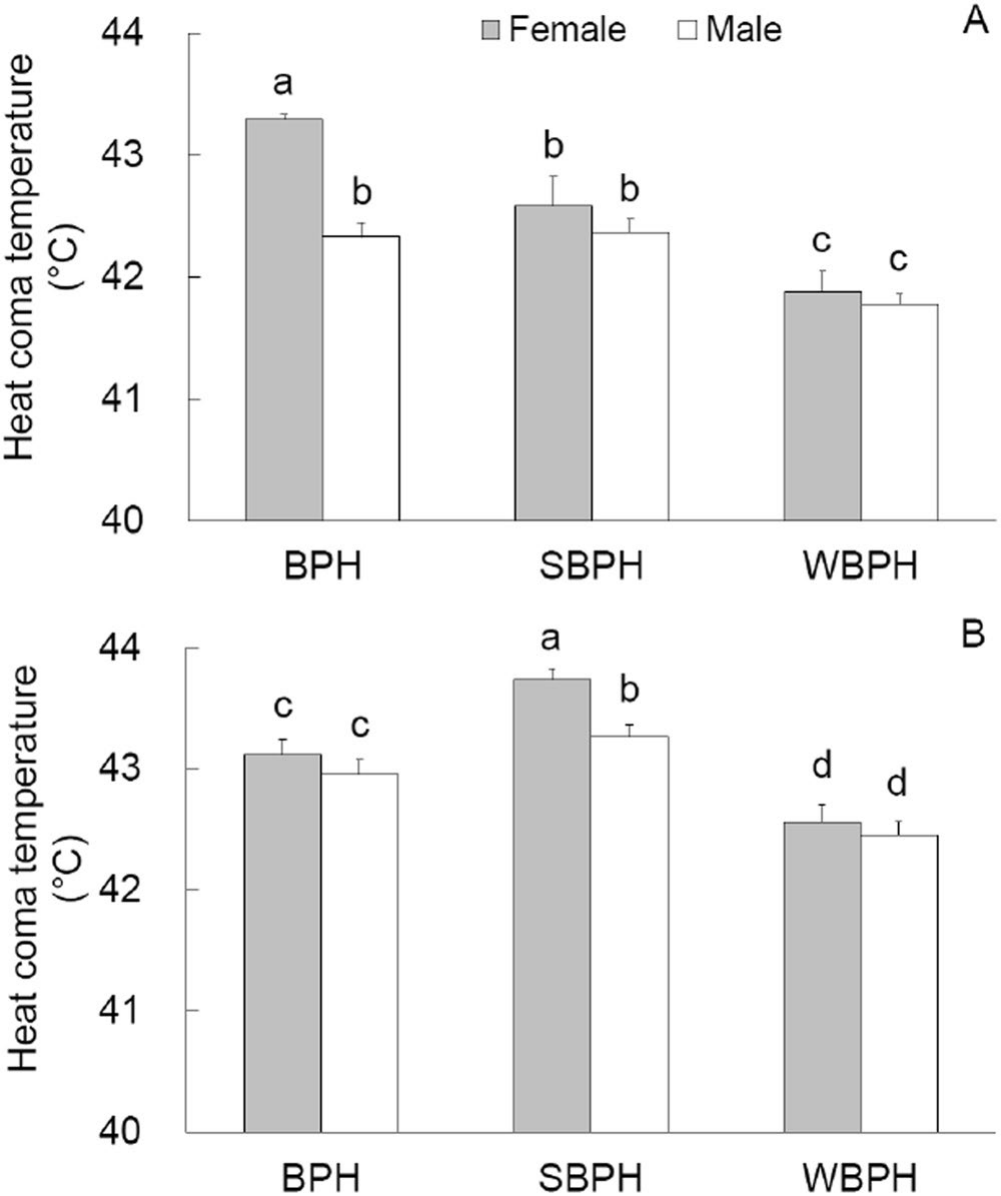
Heat coma temperature (mean ± SE) of adults of the three planthopper species measured in the absence (**A**) and in the presence (**B**) of host plant. n = 40.

Considered by sex and species of the planthopper adults, the average HCT ranged between 41.5–43.5 °C in the absence of plant material (Fig. 3A) and between 42.5–44.0 °C in the presence of plant material (Fig. 3B). Among the planthopper species, HCT differed significantly (*χ*^2^ ≥ 11.007, *P* ≤ 0.004), and was lower in WBPH than in both BPH (*Z* ≥ 2.396, *P* ≤ 0.017) and SBPH (*Z* ≥ 2.281, *P* ≤ 0.001), irrespective of sex and presence of plant material. The mean HCT values were also lower in BPH than SBPH in plant presence for both sexes (*Z* ≥ 2.31, *P* ≤ 0.021) but in plant absence, lower in females of SBPH than BPH (*Z* = 3.147, *P* = 0.002).

Significantly higher HCT values were observed in the presence than in the absence of plant material in each combination of planthopper sex and species (*Z* ≥ 3.474, *P* ≤ 0.001) except female BPH (*Z* = 1.275, *P* = 0.202). Between the sexes, BPH females in the absence of plant material and SBPH females in the presence of plant material showed higher HCT than their respective male counterparts (*Z* ≥ 4.027, *P* ≤ 0.001).

### ULT

**Figure 4:**
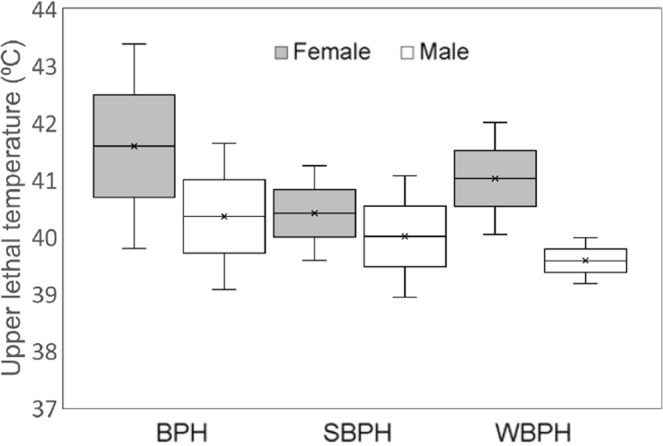
Upper lethal temperatures (ULT_50_, °C) for adults of the three planthopper species measured at a series of six temperatures with four replicates of 15 insects. The bars indicate 95% confidential limits.

ULT_50_ was detected when the insects were exposed to a series of high temperatures ranging from 37.5 to 45.0 °C (Fig. 4). There were no significant differences in ULT_50_ between the planthopper species shown by the overlapping of the 95% confidence limits. Between the sexes, WBPH females (95% confidence limits 40.04–42.01 °C) showed significantly higher ULT_50_ than males (39.18–39.99 °C).

## Discussion

Temperature is a pivotal abiotic component that significantly affects ectotherms. It influences the life history traits and physiology of insects^19^, and is an important factor determining the geographical distribution and richness of insects^3–5^, especially under climate change scenarios^20^.

Host plant contact can influence heat tolerance traits of herbivores. In the three planthopper species, CT_max_ and HCT measured in the presence of plant material were higher than in the absence of plant material. In a similar design, Alford *et al*.^16^ observed lower CT_min_ in three aphid species in the presence than in the absence of plant material and proposed that performing the measurement in the absence of plant material could result in underestimation of thermal tolerance. Host plant contact may contribute to herbivore tolerance to extreme temperatures in that: (1) an herbivore can evade thermal stress through movement in/on the host plant^5^; (2) feeding from the host plant by phloem feeders can add to their thermal tolerance. Alford *et al*.^16^ argued that phloem sap is rich in cryoprotectant carbohydrates and continuous feeding under low temperatures can enhance aphid supercooling ability and thus lower their CT_min_. When exposed to high temperatures, the accumulated polyols^21^ and glucose, protein and glycerol^22^ can stabilize protein structure against thermal denaturation; (3) feeding during exposure to high temperatures can also help maintain a water balance against dehydration through ingestion of food and water^23^. Insect dropping-off at extreme temperatures is a result of loss of locomotion coordination^24,25^. Planthoppers dropped more in the absence than in the presence of plant material at high temperatures or dropped by 50% at a lower temperature in the absence than in the presence of plant material, which further indicates that host plant contact can enhance insect’s heat tolerance. Although lack of traction on the glass tube may contribute to the insect dropping-off, the insects do drop off only when they lose locomotion coordination.

CT_max_ also differ between insect species, as shown in three *Cetoniidae* species^26^. In the cicada *Magicicada cassini*, CT_max_ is 43 °C^27^. The three planthopper species differed significantly in the upper sublethal temperatures. The planthopper adults became immobilized at 37.5–39.5 °C in the absence of plant material; this corresponds to a previous report of CT_max_ of 38 °C for BPH adults^8^. Irrespective of sex and presence of plant material, the CT_max_ values were higher in SBPH than in both BPH and WBPH and the HCT values were higher in both BPH and SBPH than in WBPH, indicating that SBPH and BPH are more heat tolerant than WBPH. These results correspond to an early report of longer lethal time in SBPH than in WBPH and BPH^12^. Because the insects were reared for six generations in the laboratory, a direct effect of sampling locations on heat tolerance can be excluded; instead, the high heat tolerance in SBPH may be adaptive to the relatively hot summer in the temperate regions where SBPH principally occurs. Additionally, SBPH is small in size (with body length of 3.33 to 4 mm) in comparison with BPH and WBPH (3.7 to 5 mm)^28^, which can add to the high heat tolerance in SBPH^8^. Since CT_max_ represents the effective limits to coordinated movement^7^, it can have important ecological significance for insect behavior^8^.

Extreme high temperature can cause mortality in insects. Fifty percent of the planthopper adults did not survive exposures of only 3 min at 39.5–41.5 °C. Similarly, approximately 50% of BPH adults were killed during 2 to 6 min exposures to 42.5 °C^8^. The current ULT_50_ values in the planthoppers are comparable to those in insects of other orders. In the tsetse fly *Glossina pallidipes*, ULT_50_ ranged between 35–38 °C during exposures of 1 to 3 h^19^ and in the adult codling moth *Cydia pomonella*, it was 44 °C following a 2 h exposure^29^. Denaturizing of proteins, enzyme structure modification and desiccation are proposed as the causes for insect death at high temperatures^9,30^. However, ULT_50_ did not differ significantly between the three planthopper species.

Insect stages and sexes may show differential vulnerability to high temperatures. In the present study, females were generally more heat tolerant than males. This may be due to the fact that male planthoppers are smaller in size and volume than females. Previous reports show that the gain and loss of heat are proportionate to the ratio of surface area to volume^8^ that is usually larger for small insects than for large insects; therefore, the planthopper males may have gained more heat in exposure to increasing high temperature than the females and thus showed lower heat tolerance than the females. The sexual difference in heat tolerance may also result from their physiological difference in response to high temperatures, which may involve the accumulation of sugars^21^ and transcriptional variation^31^. While constant high temperature induces up-regulation of most heat shock protein-related genes in both sexes, the expression of *Hsp25*.*4* and *DnaJ5* are down-regulated in the male fat body of *Bombyx mori*^31^.

The heat tolerance determined in the three planthopper species is of significance in predicting their population dynamics. The heat tolerance measured in the presence of plant material is more ecologically relevant and thus can provide results of higher relevance to the natural conditions^16^. Although field temperatures rarely reach the thermal limits gained in this study, temperatures as high as 40 °C or more occur and usually last for several hours daily during heat waves^7^. Despite the measurement of heat tolerance with the presence of plant materials in this study, caution has to be exercised in applying the data in actual pest population prediction, because the microclimates experienced by the insects in the fields, especially pre-exposure to temperature extremes or fluctuating temperatures, can greatly affect insect responses to high temperatures. Nevertheless, our results of heat tolerance echo the population dynamics of the three planthopper species. The average peak densities of SBPH and BPH in the 21th century is almost double that of the 20th century^10^. Yield losses due to SBPH damage at heading stage reached 10–20%, which never occurred in the 20th century^32^. It can be expected that the heat-tolerant insect pest species will better adapt to temperature increases associated with global warming in the future.

## Methods

### Insect cultures

Stock culture of the planthoppers (SBPH, BPH and WBPH) were established from samples collected from Zhengzhou (N34°53′35.70″, E113°38′8.53″; SBPH) and Xing’An (N25°36′13.10″, E110°41′17.30″; BPH and WBPH), China and reared on potted rice seedlings (var. Taichong1) at tillering stage in insect-proof cages. Insect cultures were housed in an environment-controlled insectary at 26 °C and LD16:8 h photoperiod. Prior to use in the experiment, the three planthopper species had been reared for 6 generations.

### Determination of critical thermal maximum (CT_max_)

CT_max_ of the three planthopper species was measured using a double-walled glass column (Weber column, height 20 cm and diameter 16 cm) connected to a programmed alcohol bath (Ministat 230-cc-NR, Huber Ltd., Germany). The double-walled glass column was similar to that used by Powell and Bale^33^. A group of 10 male or female adult planthoppers (3 day old) collected directly from the insectary into a glass tube (10 cm high, 2.5 cm diameter) covered with gauze was transferred into the Weber column preset at 26 °C. The column was subsequently closed with a plastic lid to minimize airflow and maintain a stable thermal environment and to prevent condensation. Uniformity of temperature in the column has previously been reported^16^. Before the programmed temperature increase, the planthoppers were allowed to recover at 26 °C for 30 min from handling. The observation was repeated in 4 replicates separately for males and females of each of the three planthopper species.

The temperature within the column was programmed to increase from 26 °C to 35 °C at 0.5 °C/min and then from 35 °C to 50 °C at 0.1 °C/min^8^. Temperature measured by a thermocouple within the glass tube was recorded by software (JULABO easy temp. professional) and insect movement at 11 temperatures, by a high efficiency video camera (Panasonic, HDC-HS700 DV). The video was played back for determination of CT_max_: the temperature at which an insect exhibits uncoordinated movement and drops to the bottom of the glass tube and becomes immobile^7^. It was observed that most insects were on the glass tube wall after recovery from handling and when temperature increased to 34 °C, the insects were all clinging to the glass tube. The temperatures that the insects experienced in the glass tube were the designated temperatures.

CT_max_ was also determined in the presence of plant material to provide conditions that are relevant to the field conditions as above. The difference was that the 10 adult planthoppers were aspirated onto three segments of rice stem (about 8.5 cm in height) secured with roll paper in the glass tube (10 cm high, 2.5 cm diameter). After a 30 min recovery from handling, the insects were monitored by the video camera.

### Determination of heat coma temperature (HCT)

HCT was determined using the same Weber column as for CT_max_. The temperature within the column was also initially set at 26 °C. The procedures were largely the same as that for CT_max_ measurement except that the insects were individually placed in cellular holes (diameter 1.2 cm) of a transparent plastic plate (height 10 cm, width 12 cm) within the column and allowed to settle for 30 min before temperature increase. After the temperature treatment as above, the video was watched to record the temperatures at which a planthopper last walked and moved its legs and antennae^7^.

To provide field relevant conditions, HCT was also determined for the insects in the presence of plant material as above, where the planthoppers were individually aspirated onto three segments of rice stem (about 8.5 cm in height) in a glass tube. Four replicates were exercised separately for males and females of each of the three planthopper species.

### Determination of upper lethal temperature (ULT)

ULT_50_ of the three planthopper species was determined for males and females separately. Preliminary experiments were conducted to find out the temperature range that could result in zero to 100 percent mortality. To determine ULT_50_, a group of 15 insects within a 0.9-ml Eppendorf tube was exposed to the temperatures (37.5, 39.0, 40.5, 42.0, 43.5, and 45.0 °C) generated with the programmable alcohol bath. The temperature in the bath was increased from 26 °C to a target temperature at 0.5 °C min^−1^ as above and maintained there for 3 min. After the heat stress exposure, the bath temperature was decreased back to the rearing temperature (26 °C) at the same rate as for warming. Thereafter, the planthoppers were transferred to a recovery glass tube (4 cm × 40 cm) containing rice plants (25 days old) and allowed to recover at 26 °C. Survival was assessed at 72 h after exposure. Four replicates were performed for each combination of species and sex at each of the high temperatures.

### Data analysis

Data from each replicate were averaged and SEs were calculated. The CT_max_ data were subjected to General Linear Model for significant influence of planthopper species, planthopper sex and presence of plant material, and mean difference between the planthopper species were separated by Tukey test. HCT data were subjected to Kruskal Wallis Test for significant effects of planthopper species, planthopper sex and presence of plant material individually due to equal variance could not be assumed in homogeneity analysis. When there was a significant effect, the differences were tested by Mann-Whitney U Test. Data of planthopper behaviors (dropping-off or remaining on the glass tube wall or the rice stem) were analyzed using a Pearson Chi-Square test in Crosstabs to determine if the proportion of dropping-off individuals differed between the test arena or the species. In the multiple comparisons, the tests were adjusted using the Bonferroni correction. Further, the cumulative proportions of insects dropping were regressed with temperatures using logistic estimation and the temperatures at which 50% insects dropping were calculated. The temperature resulting in 50% of mortality (ULT_50_) was determined by probit analysis using the binomial distribution. Significant difference in ULT_50_ was based on non-overlap between the 95% confidence limits^34^. All the data analysis was performed using SPSS 16.0 (SPSS Inc., Chicago, USA).

## Supplementary information

supplenentary information
