# Supplementary material for: Comparison of upper sublethal and lethal temperatures in three species of rice planthoppers
Source: Sci Rep. 2019 Nov 7;9:16191. doi: 10.1038/s41598-019-52034-7 (PMC6838088; doi:10.1038/s41598-019-52034-7)
Supplement: Supplementary file 1 — supplenentary information [file 41598_2019_52034_MOESM1_ESM.docx]

**Comparison of upper sublethal and lethal temperatures in three species of rice planthoppers**

**Shahbaz Ali^1^, Pei Li^1^, Asad Ali^1^, and Maolin Hou^1,2,3^**

^1^ State Key Laboratory for Biology of Plant Diseases and Insect Pests, Institute of Plant Protection, Chinese Academy of Agricultural Sciences, Beijing 100193, China; ^2^ Scientific Observing and Experimental Station of Crop Pests in Guilin, Ministry of Agriculture, Guilin 541399, China; ^3^ Southern Regional Collaborative Innovation Center for Grain and Oil Crops in China, Changsha 410128, China

All data generated or analysed during this study are included in this published article.
